# Identification of a novel missense *GLRA1* gene mutation in hyperekplexia: a case report

**DOI:** 10.1186/1752-1947-8-233

**Published:** 2014-06-26

**Authors:** Emese Horváth, Katalin Farkas, Ágnes Herczegfalvi, Nikoletta Nagy, Márta Széll

**Affiliations:** 1 Department of Medical Genetics, University of Szeged, Somogyi utca 4, H-6720 Szeged, Hungary; 2 Dermatological Research Group of the Hungarian Academy of Sciences, University of Szeged, Korányi fasor 6, H-6720 Szeged, Hungary; 3 Heim Pál Children’s Hospital, Üllői út 86, H-1089 Budapest, Hungary

**Keywords:** Stiff-baby syndrome, Hereditary hyperekplexia, *GLRA1* gene, Missense mutation

## Abstract

**Introduction:**

Hereditary hyperekplexia is a neurological disorder characterized by excessive startle responses with violent jerking to noise or touch, stiffening of the trunk and limbs, clenching of the fists and attacks of a high-frequency trembling. Hyperekplexia has a heterogeneous genetic background with several identified causative genes and demonstrates both dominant and recessive inheritance. Mutations in the *glycine receptor alpha 1 subunit* gene occur in about 30 percent of hyperekplexia cases.

**Case presentation:**

In this study, we report the case of a Hungarian boy whose abnormal movements, muscle stiffness and convulsions were first noted when he was 4 days old. Neurological and electrophysiological investigation suggested the clinical diagnosis of hyperekplexia.

**Conclusions:**

Direct sequencing of the coding regions and the flanking introns of the *glycine receptor alpha 1 subunit* gene revealed a novel heterozygous missense mutation (c.211A/T, p.Ile71Phe). Genetic screening of our patient’s family revealed that the clinically unaffected parents and sister do not carry the mutation, suggesting that the identified sequence change is a *de novo* mutation. Since hyperekplexia can have severe consequences, including sudden infant death due to laryngospasm and cardiorespiratory failure, identification of the causative genetic alteration(s) of the disease is high priority. Such knowledge is necessary for prenatal diagnosis, which would allow informed family planning and greater parental sensitivity to hyperekplexia 1-associated risks.

## Introduction

Hereditary hyperekplexia (HKPX, ORPHA3197) is an early-onset neurological disorder characterized by excessive startle responses with violent jerking to sudden, unexpected auditory or tactile stimuli [1,2]. Hyperekplexia usually develops shortly after birth: neonates have prolonged periods of stiffness, clenching of the fists and attacks of a high-frequency trembling [1,2]. Hyperekplexia can have severe consequences such as sudden infant death due to laryngospasm and cardiorespiratory failure [1]. The symptoms tend to resolve after infancy; however, adults may have increased startle-induced falls or nocturnal muscle jerks [1].

Hyperekplexia has a heterogeneous genetic background [3,4]. Different mutations in several genes involved in glycinergic neurotransmission can lead to hyperekplexia, and the disease exhibits both autosomal recessive and dominant inheritance [3,4]. Mutations in the *glycine receptor alpha 1 subunit* gene (*GLRA1*) result in hyperekplexia 1 (OMIM149400) and occur in about 30 percent of hyperekplexia 1 cases [5]. Mutations in other genes such as the *glycine receptor beta subunit* gene (*GLRB*; HKPX2, OMIM614619) [6], the *glycine transporter solute carrier family 6 member 5* gene (*SLC6A5*; HKPX3, OMIM614618) [7], the *glycine receptor locator gephyrin* gene (*GPHN*; OMIM603930) [8] and the *postsynaptic glycine enhancer collybistin* gene (*ARHGEF9*; OMIM300429) have also been associated with this clinical condition [9].

In this manuscript, we present the clinical and genetic investigations of a Hungarian family affected by hyperekplexia 1 and the identification of a novel disease-causing heterozygote missense mutation of the *GLRA1* gene.

## Case presentation

A male neonate was born to term at the 40^th^ week of gestation by cesarean section delivery after an uneventful pregnancy. His birth weight was 3990g and Apgar score was 9/10. At day 1 post-term, he developed a pneumothorax and was admitted to the perinatal intensive care unit for extra oxygen and parenteral fluid therapy. At day 4 post-term, abnormal movements, stiffness of the muscles and convulsions were observed, and phenobarbital therapy was initiated. A neurogical investigation described dyskinesia. At day 11 post-term, he was hospitalized in a developmental neurology ward. An examination did not identify any hypoxia-induced regulatory abnormalities. The observed recurrent muscular hypertonia was attributed to a suspected ion channel disorder and carbamazepine therapy was initiated.

Ultrasonography of his hip indicated the possibility of dysplasia on the left side, and ultrasonography of his abdomen revealed bilateral mild pyelectasis. The results of neurosonography, electroencephalography and magnetic resonance imaging of the head did not indicate any abnormalities of the central nervous system.

**Figure 1:**
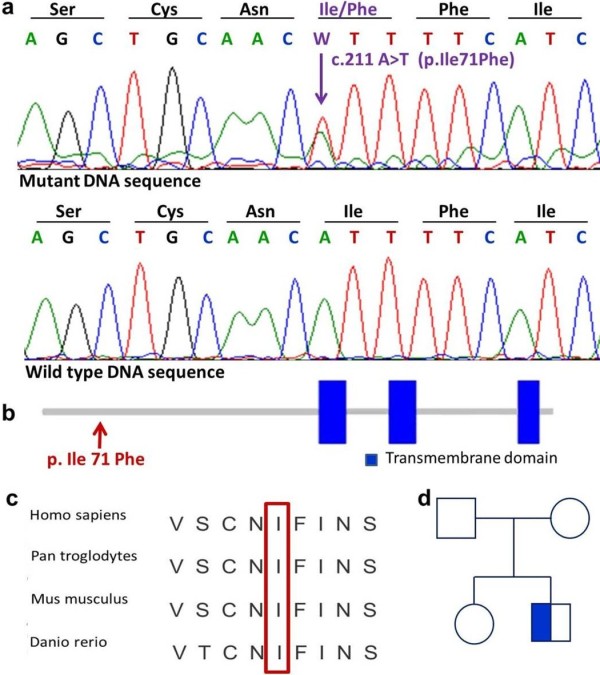
**Identification of a novel mutation in the *GLRA1 *gene and genetic screening of the affected family. (a)** Direct sequencing of our patient (II/2) DNA revealed a novel heterozygous missense mutation in exon 3 of the *GLRA1* gene. **(b)** The clinically unaffected family members (I/1, I/2 and II/1) carry only the wild-type sequence, suggesting a *de novo* mutation. **(c)** Comparison of GLRA1 protein sequences in the region of the mutation (p.Ile71Phe) from different species indicates that the region is highly conserved. **(d)** Genetic analysis of the affected family suggests that the identified sequence alteration is a *de novo* mutation.

After pre-test genetic counseling was performed and written informed consent was obtained from his parents, a blood sample was taken from our patient, his clinically unaffected sister and his clinically unaffected parents for genetic investigation. Genomic deoxyribonucleic acid (DNA) was isolated using a BioRobot® EZ1 DSP Workstation (Qiagen; Hilden, Germany). The coding regions of the *GLRA1* gene and the flanking introns were amplified and sequenced (using primer sequences obtained from the UCSC Genome Browser, http://www.genome.ucsc.edu/). A molecular genetic investigation was carried out by the amplification and direct sequencing of the *GLRA1* gene. Having identified the putative causative mutation in our patient (II/1), mutation screening of the unaffected family members (I/1, I/2 and II/1) was carried out. Direct sequencing of the coding regions and the flanking introns of the *GLRA1* gene revealed a novel heterozygous missense mutation (c.211A/T, p.Ile71Phe) in exon 3 (Figure 1a). Mutation was confirmed by a second validation method, re-sequencing. Genetic screening of the affected family revealed that the clinically unaffected parents (I/1 and I/2) and the unaffected sister (II/1) did not carry the mutation, suggesting that the identified novel sequence alteration is a *de novo* mutation in our patient (Figure 1d).

## Conclusions

In this manuscript, we report the case of a Hungarian patient with hyperekplexia, a potentially fatal neurological disorder characterized by pronounced startle responses. Abnormal movements, stiffness and convulsions were first noted in our patient at day 4 post-term, which correlates well with the early onset of the disease.

Hyperekplexia has been linked to genetic alterations in genes involved in an inhibitory neurotransmitter, glycine neurotransmission [3,4]. Both compound heterozygous patients and homozygous mutation carriers have been described in the literature for recessive forms of the disease [10]. A heterozygous missense mutation (c.211A/T, p.Ile71Phe) was detected in our patient in exon 3 of the *GLRA1* gene, establishing the diagnosis of hyperekplexia 1 and suggesting that the mutation is an autosomal dominant form of the disease.

The *GLRA1* gene encodes a neurotransmitter-gated ion channel transmembrane protein with three transmembrane segments (TM1-3) [11,12]. Binding of glycine to its receptor increases the chloride conductance, produces hyperpolarization and, thus, the inhibition of neuronal firing [11,12]. Previous studies have attributed dominant forms of hyperekplexia 1 to mutations within the pore-lining transmembrane segment (TM2) and adjacent regions, recessive forms to mutations within the other transmembrane segments (TM1 and TM3), and the null allele of the *GLRA1* gene to the deletion of exons 1-7 [11,12]. The novel heterozygous missense mutation (p.Ile71Phe) reported here is located close to the NH_2_-terminal of the GLRA1 protein outside the transmembrane segments (Figure 1b), in a highly conserved region (Figure 1c). Other missense mutations have been detected in this region in patients with hyperekplexia 1 (p.Trp68Cys and p.Arg72His) [3], and in spasmodic mice (p.Ala52Ser) [13]. The functional analysis performed on the spasmodic mouse model suggested that the p.Ala52Ser missense mutation results in reduced glycine sensitivity [13]. Recessive mutations in the N-terminal regions or majority of recessive hyperekplexia mutations have trafficking defects [14,15]. Dominant mutations can cause reduced glycine sensitivity [14,15]. Based on these previous studies, we hypothesize that the reported novel missense mutation detected in our Hungarian patient might lead to reduced glycine sensitivity as well. It is also interesting to note that previously reported missense mutations in this region (human p.Trp68Cys and p.Arg72His and murine p.Ala52Ser) are all associated with the recessive form of hyperekplexia 1, indicating the possibility that other undetected mutations might contribute to the clinical symptoms of our Hungarian patient.

Our patient’s clinically unaffected family members were also screened and shown to carry only wild-type sequence of the *GLRA1* gene. Our results suggest that this novel missense sequence change (c.211A/T, p.Ile71Phe) identified in the index patient is a *de novo* mutation.

The consequences of the hyperekplexia 1 can be severe, warranting further efforts to elucidate the nature of the disease despite the complications implicit with the heterogenic genetic background. With the identification of the underlying genetic abnormalities, prenatal screening is available for affected families and allows informed family planning. In the future, knowledge of the genetic causes of this life-threatening disease may also contribute to the development of novel therapeutic alternatives.

## Consent

Written informed consent was obtained from the patient’s parents for publication of this case report and any accompanying images. A copy of the written consent is available for review by the Editor-in-Chief of this journal.

## Abbreviations

HKPX: hereditary hyperekplexia; *GLRA1*: *glycine receptor alpha 1 subunit* gene.

## Competing interests

The authors declare that they have no competing interest.

## Authors’ contributions

EH, KF, ÁH, NN, MS met the International Committee of Medical Journal Editors (ICMJE) criteria for authorship. EH contributed to data collection and the first draft of the manuscript. KF and NN carried out the mutation analysis. ÁH cared for the patient. MS was a mentor who contributed equally to this work. All authors read and approved the final manuscript.
